# Adipokines and Aging: Findings From Centenarians and the Very Old

**DOI:** 10.3389/fendo.2019.00142

**Published:** 2019-03-14

**Authors:** Yasumichi Arai, Kei Kamide, Nobuyoshi Hirose

**Affiliations:** ^1^Center for Supercentenarian Medical Research, Keio University School of Medicine, Tokyo, Japan; ^2^School of Allied Health Sciences, Osaka University Graduate School of Medicine, Osaka, Japan

**Keywords:** centenarian, longevity, adipokines, adiponectin, frailty

## Abstract

Adipose tissue, which was once considered as a simple energy storage depot, is now recognized as an active endocrine organ that regulates the whole-body energy homeostasis by secreting hundreds of bioactive substances termed adipokines. Dysregulation of adipokines is a key feature of insulin resistance and a metabolic syndrome associated with obesity. Adipokine dysregulation and insulin resistance are also associated with energy-deprivation conditions, such as frailty in old age. Previous studies have demonstrated that preserved insulin sensitivity and low prevalence of diabetes are the metabolic peculiarities of centenarians, suggesting the possible role of adipokine homeostasis in healthy longevity. Among the numerous adipokines, adiponectin is regarded as unique and salutary, showing negative correlations with several age- and obesity-related metabolic disturbances and a positive correlation with longevity and insulin sensitivity among centenarians. However, large-scale epidemiological studies have implied the opposite aspect of this adipokine as a prognostic factor for all-cause and cardiovascular mortality in patients with heart failure or kidney disease. In this review, the clinical significance of adiponectin was comparatively addressed in centenarians and the very old, in terms of frailty, cardiovascular risk, and mortality.

## Introduction

Advances in obesity research from the early 1990s have shed light on the prominent role of adipose tissues as an active endocrine organ that regulates energy homeostasis by secreting bioactive substances termed adipokines (1). A growing number of these adipokines have been identified, and their roles in regulating whole-body energy homeostasis via modulation of several signaling cascades in the target tissues are being increasingly discovered. Dysregulation of adipokines is regarded as a key feature of insulin resistance, hyperglycemia, and dyslipidemia, as well as the comorbidities of obesity, such as metabolic syndrome, type 2 diabetes mellitus (T2DM), and cardiovascular disease (2, 3). However, accumulating evidence signifies that adipokine dysregulation is also associated with wasting syndromes such as cachexia and sarcopenia, suggesting that adipose endocrine function is essential for maintaining whole-body energy homeostasis, which is indispensable for a multitude of physiological functions under the conditions of both energy excess and deprivation (4). Furthermore, genetic manipulation of the adipose tissue has been shown to promote longevity in mice models, denoting its possible role in regulating the lifespan (5).

Centenarians have been able to delay the onset of life-threatening diseases, such as cardiovascular diseases or cancers, or even escape from them altogether until the late years of life, thus serving as models for healthy aging (6, 7). For more than three decades, centenarian studies have been conducted to identify biological markers conducive to healthy longevity. Several key pathways for maintaining health and longevity have been thereby discerned; of which insulin sensitivity has been recognized as one of the major pathways to healthy longevity, which is conserved right from dwarf mice to centenarians (8). In this review, we have discussed the possible roles of adipokines, especially adiponectin, in regulating longevity in humans and the possibility that this regulation may be mediated via the preservation of insulin sensitivity and compensatory mechanisms against inflammation and oxidative stress that occurs with aging.

## Insulin Sensitivity as a Hallmark of Longevity: Lessons From Long-Lived Mice and Centenarians

Caloric restriction is one of the most replicated pro-longevity interventions across species (9). Interestingly, calorie-restricted mice and a series of long-lived rodent models, such as the Ames dwarf, Snell dwarf, and growth hormone receptor knockout, share common features, including reduced GH/insulin-like growth factor 1 (IGF-1) signaling, preserved insulin sensitivity, reduced growth, and body size (10). The precise molecular mechanism by which reduced somatotropic signaling enhances longevity has not yet been completely elucidated; however, downregulation of reactive oxygen species and increased stress resistance may be involved in the aging delay witnessed in these models (11–13). In humans, insulin sensitivity normally decreases during aging; nonetheless, accruing evidence has documented the preservation of insulin sensitivity and glucose homeostasis among the centenarians and their offspring. In the late 1990s, Paolisso et al. first reported that glucose tolerance and insulin sensitivity were better preserved in healthy centenarians than in elderly individuals aged >75 years using a euglycemic glucose clamp method (14). Subsequently, in the Leiden longevity study, Wijsman et al. revealed that the offspring of long-lived siblings had better insulin sensitivity than the controls of corresponding age and body mass index (BMI), hinting at the inheritable component of insulin sensitivity and longevity (15). Metabolic syndrome (MS) and T2DM, both of which are devastating consequences of insulin resistance, increase in older adults (16, 17). Intriguingly, the low prevalence of these metabolic diseases is reportedly observed worldwide among the centenarians. In the Tokyo Centenarian Study, Takayama et al. examined 304 centenarians living in the Tokyo metropolitan area and inferred that the prevalence of diabetes mellitus was only 6.0%, which is less than half of that in the general population of 60s (15.3%) and 70s (14.7%) in Japan (18). The Finnish Centenarians Study (19) presented a 10% prevalence of T2DM among the Finnish centenarians, which was lower than that recorded among the 65- to 85-year-old Finnish individuals. Similarly, the Italian Multicenter Study on Centenarians (20) demonstrated that 4.9% of the 602 centenarians had T2DM, and the New England Centenarian Study stated that 4% of the 424 centenarians had T2DM (21), both of which were lower upon comparison with the respective aged but younger populations. These findings collectively indicate that preserved insulin sensitivity and glucose homeostasis are the hallmarks of longevity in both rodents and humans.

## Adipokine Profiles of Centenarians

To date, vigorous basic research has been conducted on the biology underlying the association between insulin sensitivity and longevity, and the adipokines have emerged as a possible mechanistic link (22, 23). Among these substances, adiponectin is one of the most potent molecules regarding insulin sensitizing activity. Unlike the majority of adipokines, plasma adiponectin levels displayed an inverse correlation with adiposity and are reduced in obese individuals (24). Adiponectin plays an anti-diabetic role within the liver and skeletal muscles by facilitating the glucose uptake at these sites, thereby enhancing the insulin sensitivity. Adiponectin also has anti-inflammatory and anti-atherogenic properties and is thus regarded as an immensely beneficial adipokine (25). Leptin is another adipokine of interest that regulates whole-body energy homeostasis by restricting food intake and stimulating energy expenditure (26). In a series of rat models, decreased visceral fat mass, obtained either by caloric restriction or surgical resection, improved age-related insulin resistance, possibly via alteration of leptin and other adipokine secretions (27, 28). Moreover, mice with fat-specific disruption of the insulin receptor gene (FIRKO) have been demonstrated to exhibit reduced adiposity, lower fasting insulin levels, and enhanced longevity (5). FIRKO mice were also characterized by elevated serum adiponectin levels. These rodent models demonstrated that reduced adiposity itself can extend the lifespan and altered adipokine secretion, especially the upregulation of adiponectin and insulin sensitivity, may be the critical mediators of this process.

On the basis of these experimental evidences from longevity model animals, centenarian studies investigated the association between adipokines and healthy longevity in humans. We used PubMed to search for relevant publications before November 2018 in English. We used the search terms “centenarians” by title/abstract screening and “adipokines,” “adipocytokines,” “leptin,” and “adiponectin.” We also checked the reference lists of the relevant publications identified in the search. We excluded articles without control groups (usually healthy, older adults), and identified seven studies as shown in Table 1. In the first study of its kind, Paolisso et al. demonstrated that the plasma leptin levels were higher in the 19 healthy centenarians than in adults aged <50 years, but lower in elderly aged 75–99 years (29). The levels in healthy centenarians were inversely correlated with IGF-1/IGF-1 binding protein 3 molar ratio, alluding the possible effects of the unbound form of IGF-1 on circulating leptin regulation (29). In contrast, Baranowska et al. reported that 75 female centenarians had significantly lower leptin levels than elderly females aged 64–67 years or younger females aged 20–43 years (30). Low leptin levels in centenarians seem to be independent of BMI or fat mass, because BMI of centenarians did not differ from that of younger females. While Pareja-Galeano et al. demonstrated that 81 healthy centenarians without major disease had significantly higher leptin levels than sex-matched elderly controls aged 70–80 years, although BMI was not compared between the two groups (31). Recently, in older adults, Lana et al. demonstrated that higher leptin levels were associated with a greater risk of incident frailty, which was independent of body fat, homeostasis model assessment for insulin resistance (HOMA-IR), or CRP (32). Conflicting findings over leptin levels in centenarians may reflect multiple regulatory mechanisms of this adipokine with aging. Regarding adiponectin, Arai et al. reported that 66 female centenarians had higher plasma adiponectin levels than the BMI-matched younger females (33). In addition, the high plasma adiponectin concentrations in centenarians were associated with an advantageous metabolic phenotype, including higher high-density lipoprotein-cholesterol (HDL-C) levels and lower hemoglobin A1c, and negatively correlated with C-reactive protein and E-selectin concentrations (33). Bik et al. also testified the occurrence of hyperadiponectinemia in Polish centenarians (34); the researchers found an inverse correlation between plasma adiponectin levels and HOMA-IR, a reliable marker of insulin resistance. In addition, Atzmon et al. also claimed that 118 long-lived individuals (aged ≥95 years) had increased the adiponectin levels and that the levels were inversely correlated with BMI, waist circumference, and percent body fat, but positively correlated with HDL-C and the lipoprotein particle size (35). In the circulation, adiponectin has three oligomeric forms, including a trimer (low-molecular weight), hexamer (medium-molecular weight), and high-molecular weight (HMW) form. Among them, HMW adiponectin is the major active form as it displays greater insulin sensitizing and anti-inflammatory properties in experimental studies (36). Bik et al. investigated the adiponectin isoforms in 58 Polish centenarians and found that they have significantly higher levels of total isoforms, as well as all isoforms of adiponectin individually, compared with elderly individuals aged approximately 70 years (37). The investigators also proved that both total and HMW adiponectin were positively correlated with HDL-C and negatively correlated with the fasting glucose and insulin levels, HOMA-IR, and triglycerides (37). As presented in Table 1, most studies demonstrated a high plasma adiponectin level among the centenarians, which can be correlated with a preferable metabolic phenotype, including high HDL-C and insulin sensitivity, thereby signifying the beneficial metabolic effects of this adipokine on enhancing longevity. However, because centenarian studies on circulating adiponectin are exclusively based on cross-sectional design, whether high adiponectin levels are the cause or consequence of long life remain to be elucidated.

**Table 1:** Centenarian studies reporting circulating leptin and adiponectin levels.

**References**	**Sample size no. of centenarians (% of females)**	**Controls**	**BMI**	**Leptin level**	**Adiponectin level**
Paolisso et al. (29)	19 (58% females)	30 Adults (aged <50 years) 30 elderly	↓	↔^*^	ND
Arai et al. (33)	66 (100% females)	66 BMI-matched young females	↔	ND	↑
Bik et al. (34)	22 (100% females)	45 young females 19 elderly females 36 obese females	↓	ND	↑
Baranowska et al. (30)	75 (100% females)	45 young females 26 elderly females 37 obese females	↓	↓	↑
Atzmon et al. (35)	118 (aged ≥95 years, 74% females) 228 offspring (50% females)	78 elderly	↓ (probands) ↔ (offspring)	ND	↓ (probands) ↔/^↑^†^^(offspring)
Meazza et al. (38)	48 (77% females)	50 elderly 62 neonates	↓	↓	↑
Bik et al. (37)	58 (86% females)	68 elderly	↓	ND	Total ↑, HMW ↑ MMW ↑, LMW ↑
Pareja-Galeano et al. (31)	81 (51% females)	46 elderly	ND	↑	↔

* *Leptin levels in centenarians was higher than that in adults, but lower than that in the elderly*.

↑† *Adiponectin levels in offspring was lhigher than that in elderly controls when adjusted for age, sex, and BMI. ↑ Higher in centenarians compared to controls. ↓ Lower in centenarians compared to controls. ↔ No difference between centenarians and controls*.

## Genetic Determinants of Circulating Adiponectin Levels

There are several studies on the genetic variations that determine the circulating adiponectin level. The first genome-wide linkage study asserted that the gene (*ADIPOQ*) in 3q27 was highly associated with circulating adiponectin levels in Hispanic-Americans (39). Thereafter, the most reported single nucleotide polymorphism (SNP) in *ADIPOQ*, rs266729, located in the promoter region, was significantly linked with the circulating adiponectin level. This was demonstrated because subjects with GG genotype in rs266729 exhibited higher plasma adiponectin levels than those of other genotypes in some replicated studies, including those hailing from different ethnic backgrounds (40, 41). This SNP is supposed to be the most promising genetic variation related to adiponectin level and also the risk of MS (42), T2DM (43), and insulin resistance (41). Another SNP located in the promoter region of *ADIPOQ*, rs1656930, was highly connected with the adiponectin levels of elderly Japanese subjects (44). Another genome-wide association study (GWAS) revealed that SNP (rs4783244), located in intron 1 of the T-cadherin gene (*CDH13*) was significantly associated with the plasma adiponectin levels of Taiwanese (45), Japanese (46) subjects and the risk of MS and T2DM (45). These SNPs are also implicated in cardiovascular remodeling, such as carotid intima-media thickening (40) and cardiovascular complications (47), possibly through the modulation of circulating adiponectin levels.

The association between adiponectin genotype and longevity was tested in a cohort of Ashkenazi Jews with exceptional longevity. Atzmon et al. examined the plasma adiponectin levels and *ADIPOQ* genotypes in long-lived individuals (>95 years), their offspring and controls, and uncovered that the two common variants of *ADIPOQ* were over-represented among the male long-lived individuals compared with the corresponding controls (35). Interestingly, the findings were not observed in the female participants. Further studies with a large sample size are warranted to replicate the association between *ADIPOQ* and human longevity.

## Adiponectin and Cardiovascular Mortality: Adiponectin Paradox

In contrast to the basic science reports and findings from centenarian studies, which collectively support the beneficial metabolic effects of adiponectin, accumulating observational studies have demonstrated an unexpected association between high adiponectin levels and increased mortality in patients with cardiovascular disease, particularly heart failure. In 195 patients with chronic heart failure, Kistorp et al. demonstrated that high plasma adiponectin levels were associated with increased mortality risk, independent of the severity of the heart failure and BMI (48). Moreover, circulating adiponectin was significantly correlated with N-terminal pro-brain natriuretic peptides (NT-proBNP), and the association between adiponectin and mortality remained significant after adjustment by NT-proBNP (48). Subsequently, the connection between adiponectin and mortality has been replicated in studies with much larger samples and other clinical settings, such as ischemic heart disease, type 1 and type 2 diabetes, end-stage renal disease, and even in the general elderly population (49–51). These findings are counterintuitive to its salutary metabolic effects and thus called adiponectin paradox. A meta-analysis of earlier studies, including 24 prospective studies suggested that the paradoxical association between high adiponectin levels and increased all-cause mortality risk is more significant in those with coronary heart disease (CHD) at the baseline than those without CHD (52). Sex dimorphism is also documented, and high adiponectin levels predict cardiovascular mortality in men, but not in females with T2DM (53). In contrast, recent meta-analysis, including 55 and 28 studies for all-cause and cardiovascular mortality, respectively, demonstrated that 1-SD increment of adiponectin was associated with a 24 and 28% increase in all-cause and cardiovascular mortality, respectively (54). When restricted to studies with natriuretic peptides measurement, a substantial reduction in the associations between circulating adiponectin and all-cause and cardiovascular mortality was substantially attenuated by adjustment for natriuretic peptides, denoting that the adiponectin paradox is partly mediated by natriuretic peptides (54). Interestingly, Tsukamoto et al. demonstrated that both atrial and brain natriuretic peptides enhance the production of adiponectin in adipocytes and that the intravenous infusion of ANP increases circulating adiponectin levels in humans (55). These data imply that the paradoxical association between circulating adiponectin and mortality may be indirect and mediated by coexisting cardiovascular risk factors, such as natriuretic peptides.

Another plausible mechanism underlying the paradoxical association is adiponectin resistance. Adiponectin enhances insulin sensitivity by improving glucose uptake in the skeletal muscles, inhibiting gluconeogenesis and stimulating the β-oxidation of fatty acids through adiponectin receptor 1 (Adipo R1) and receptor 2 (56, 57). In patients with chronic heart failure, Van Berendoncks et al. proved that adiponectin levels are increased, both in circulation and in their gene expression in the skeletal muscle, but also demonstrated a downregulation of Adipo R1 and deactivation of the PPAR-α/AMP-activated protein kinase pathway. Hence, increased adiponectin concentrations are not effectively connected with downstream signal transductions, resulting in functional adiponectin resistance (58). Therefore, in this context, high circulating adiponectin in heart failure represents the presence of a protective mechanism to counteract adiponectin resistance and the compromised energy metabolism.

A causal relationship between adiponectin and CHD has been addressed by genetic research. In a Mendelian randomization study, Borgers et al. examined the link between the genetic variant of adiponectin levels and CHD risk using data from GWAS consortia (59) and found no causal role of adiponectin level in CHD risk. On the other hand, Uetani et al. observed that a GWAS-based SNP in *CDH13* was associated with both circulating HMW adiponectin levels and increased all-cause mortality in the general population (60), although the researchers did not address cardiovascular-specific mortality. More experimental and epidemiological studies are needed to determine whether adiponectin has direct deleterious effects on cardiovascular pathology and mortality.

## Adiponectin and Frailty in the Very Old: Another Paradox

Paradoxical associations between high adiponectin levels and mortality are conspicuous in the very old even without cardiovascular disease or chronic kidney disease, indicating the potential involvement of this adipokine in geriatric syndrome, such as frailty and sarcopenia. This topic has been vigorously addressed in our longitudinal cohort study for old people, known as the SONIC (i.e., septuagenarians, octogenarians, and non-agenarians investigation with centenarians) study, which investigated the age differences and similarities in factors influencing healthy aging and psychological well-being, including psychological (i.e., cognition, change in emotion and compensation, personality, and psychological development); social (i.e., socio-economic status and social relationship); and medical, dental, and nutritional aspects (61). In 353 community-dwelling older adults of approximately 83 years, Nagasawa et al. deduced an association between circulating adiponectin and frailty status according to the cardiovascular health study criteria (62). The investigators found significantly higher adiponectin levels in frail subjects than in their non-frail counterparts. Moreover, a multivariate logistic regression analysis affirmed that the elevated adiponectin level, higher estimated glomerular filtration rate, and lower hemoglobin were independent determinants of the pre-frail/frail status when compared with the non-frail status. Weight loss, low muscle mass, and poor physical functioning are the core components of frailty in older adults. Among the 2,821 participants of health ABC study, who had whole-body dual-energy DXA, Baker et al. explored the independent association among circulating adiponectin, body composition, physical functioning, and mortality (63). The authors uncovered a significant relationship between high adiponectin and historical weight loss, low muscle mass, and low muscle density. Adiponectin was substantially associated with increased risk of incident disability and all-cause mortality; however, when adjusted for weight loss and physical performance at baseline, the association was attenuated and no longer significant. On the basis of these findings, the researchers suggested that the high adiponectin levels in the very old may represent a compensatory response to low energy availability in the setting of starvation. Interestingly, high adiponectin in plasma is associated with low functional capacity in patients with chronic heart failure (64), signifying that this adipokine may be a marker for wasting in CHF. Moreover, among 1,303 patients with predialysis chronic kidney disease, Hyunn et al. demonstrated that a higher adiponectin level was associated with protein malnutrition defined by hypoalbuminemia, low BMI, low urine creatinine excretion, and low protein intake (65). Collectively, these epidemiological findings suggested that circulating adiponectin may be a useful biomarker of catabolic processes, such as sarcopenia and cachexia, in the chronic conditions, which are frequently associated with weight and muscle loss as well as high mortality risk among the elderly.

## High Adiponectin Levels in Centenarians: Possible Compensatory Responses to Maintain Metabolic and Redox Homeostasis

**Figure 1:**
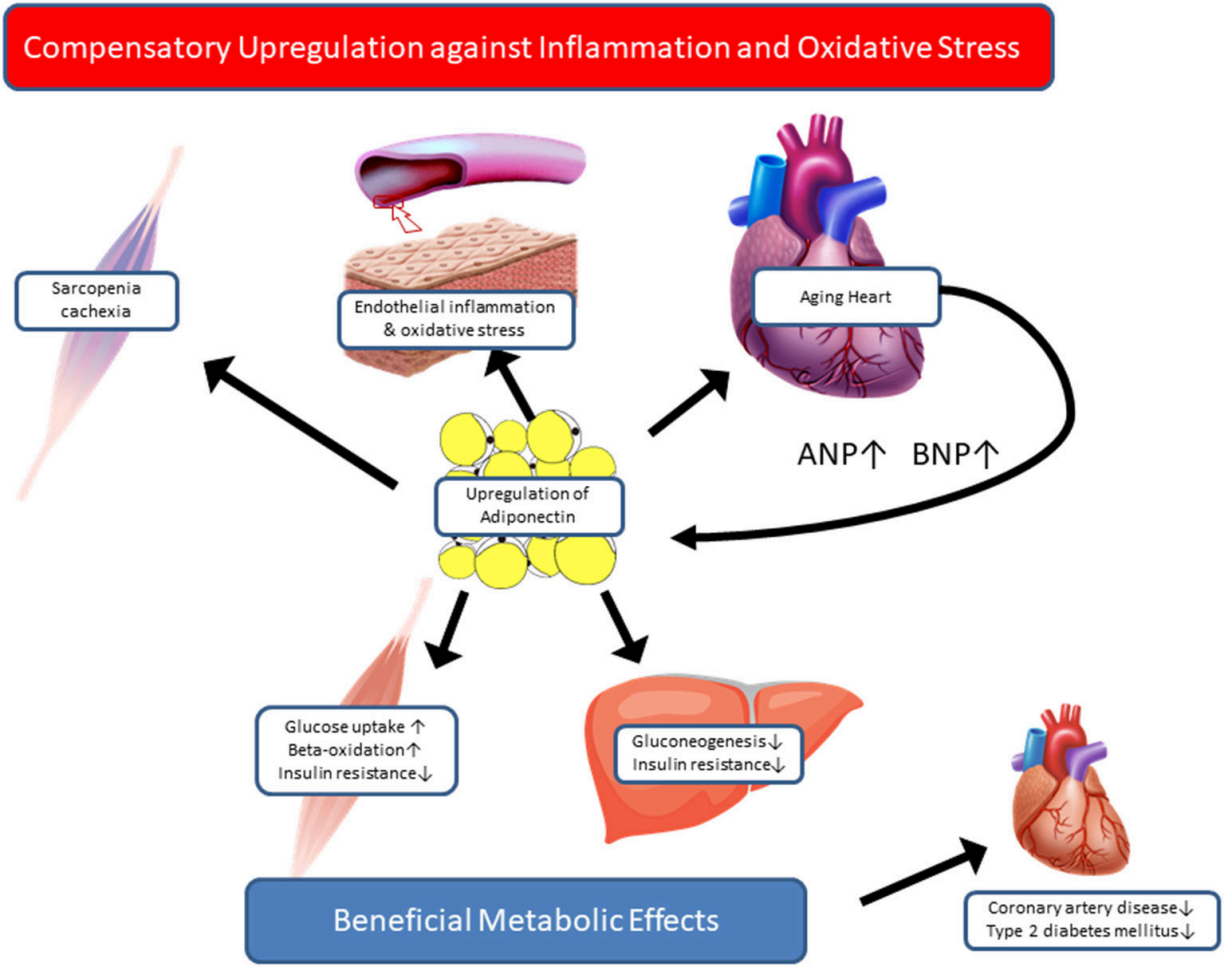
Hypothetical roles of adiponectin in centenarians. Based on paradoxical evidence regarding the association between adiponectin and health outcomes, we propose two hypothetical roles of adiponectin in centenarians. Adiponectin is a salutary adipokine that is present at high levels in healthy and lean individuals, e.g., centenarians. High adiponectin levels are associated with low insulin levels and low HOMA-IR, and a favorable lipid profile, which is consequently associated with a low risk of coronary artery disease and type 2 diabetes. In contrast, in patients with catabolic states, such as chronic heart failure and sarcopenia, adiponectin is upregulated as a part of compensatory mechanisms against inflammation and oxidative stress in relevant organs. Once the compensation fails, in case of adiponectin resistance, high adiponectin levels predict high mortality in advanced stage of disease or aging.

In contrast to the beneficial metabolic and cardioprotective effects of adiponectin observed in long-lived animal models, immense epidemiological evidence supports the paradoxical relationship between high adiponectin levels and poor outcomes in cardiovascular and geriatric conditions. If that is the case, how can we interpret the high adiponectin levels in centenarians? Most of the centenarian studies aiming at circulating adiponectin are cross-sectional and comprise a relatively small sample size, hence posing limitations in elucidating the causal relationship between high adiponectin and exceptional longevity. Recently, Sebastiani et al. assessed 38 age-related circulating biomarkers in ~5,000 healthy, older adults of the long-life family study, aged 25–110 years, and 34 biomarkers had a statistically significant association with age at assessment (66). Among these, adiponectin and NT-proBNP showed similar correlation coefficients with age (*r* = 0.3178, *p* < 0.001; *r* = 0.3793, *p* < 0.001, respectively), although correlation between these two biomarkers are not shown. Their findings suggest that the high adiponectin levels in centenarians may be the consequence of advancing age, even without prevalent cardiovascular disease. To examine prognostic significance of adiponectin, we investigated the association between a set of adipokines and all-cause mortality in a prospective cohort study of 252 centenarians, aged 100–108 years (67). In this work, we noticed the significant association of low leptin and high TNF-alpha with higher mortality risk. Interestingly, stratified analysis by BMI revealed that the significant association of leptin and mortality was reduced in lower-BMI group, suggesting that it was mediated by low fat mass. In contrast, association between TNF-alpha and mortality was increased in lower-BMI group compared to their counterparts, suggesting that catabolic states, such as sarcopenia and cachexia, contribute to high mortality in centenarians, at least in those with low BMI. However, plasma adiponectin levels were not associated with mortality in the total sample or in the lower-BMI group; thus, our results do not support the paradoxical association between high adiponectin and increased mortality in the extreme old age. Although some aspects of the complicated relationship between adiponectin and health outcomes are still unresolved, based on the findings so far, we would like to propose a hypothesis that high adiponectin levels in centenarians might reflect the compensatory response to maintain metabolic homeostasis and to counteract oxidative stress and inflammation, which are relevant in catabolic states, such as sarcopenia and chronic heart failure (Figure 1). Currently, we have extended the adiponectin study to semi-supercentenarians (individuals aged >105 years) and supercentenarians (individuals aged >110 years) with various cardiovascular biomarkers to test the hypothetical roles of adiponectin in longevity. Further longitudinal research with sequential measurements of adiponectin and other biomarkers is warranted to gain a better understanding of the role played by adiponectin in promoting healthy aging and longevity.

## Data Availability

The datasets for this study will not be made publicly available because this is not an original article, but a review summarizing previous findings.

## Author Contributions

YA and KK drafted the paper. NH provided critical review of the manuscript. All authors confirmed the final version of the manuscript.

### Conflict of Interest Statement

The authors declare that the research was conducted in the absence of any commercial or financial relationships that could be construed as a potential conflict of interest.
